# The replication rate of *Anaplasma marginale* is temperature-mediated in ticks.

**DOI:** 10.17912/micropub.biology.001442

**Published:** 2025-04-22

**Authors:** Popy Devnath, Susan M. Noh, Shelby M. Jarvis, Kayla Earls, Kennan J. Oyen

**Affiliations:** 1 Washington State University, Pullman, Washington, United States; 2 Animal Disease Research Unit, Agricultural Research Service, Pullman, WA, US

## Abstract

*Anaplasma marginale* , the cause of bovine anaplasmosis, a serious production-limiting disease of cattle found worldwide, is biologically transmitted by adult male *Dermacentor* spp. ticks in the United States. We tested the impact of 9 temperatures on infected *D. andersoni* and found that the replication of *A. marginale* in tick midguts and salivary glands is temperature dependent. There were higher bacterial levels between 32°C and 37°C than between 4°C to 26°C. We observed 100% mortality in ticks at 42°C. Future research should explore the mechanisms of temperature-dependent replication in *A. marginale* and possible links to transmission rates under climate change.

**Figure 1. The impact of temperature on A. marginale in ticks:**
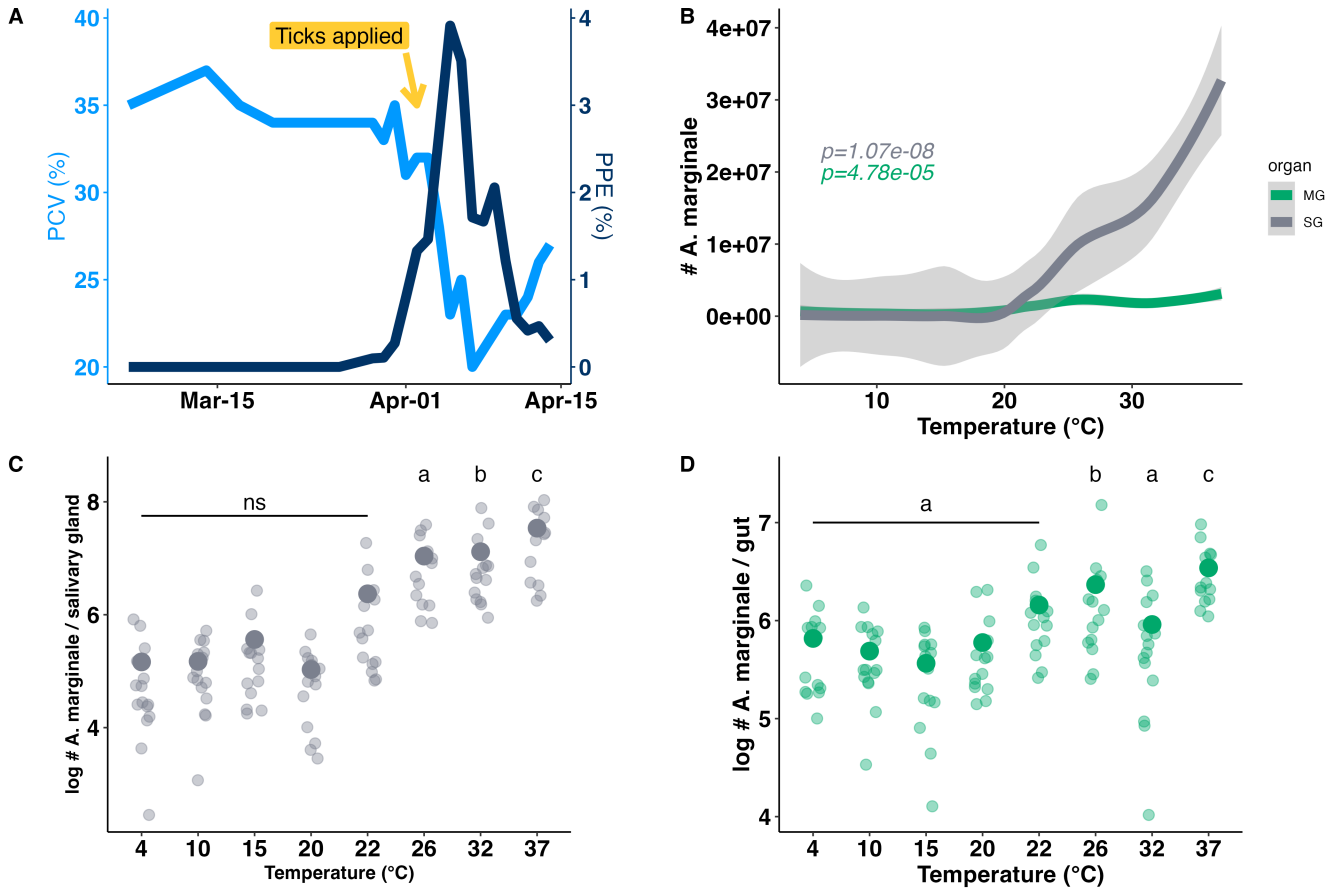
**A.** **The percent of infected erythrocytes (PPE) (navy blue) and packed cell volume (PCV) (light blue) through time:** A calf was inoculated with *Anaplasma marginale* and *Dermacentor andersoni* ticks fed for 7 days during rising parasitemia (arrow). **B. Levels of *A. marginale* increase significantly with temperature:** Smoothed curves showing the relationship between temperature (°C) and the number of *A. marginale* in tick midguts (green line) and salivary glands (grey line). **C. *A. marginale* levels in salivary glands (grey) based on quantitative PCR for *msp5* :** Infection level in salivary glands was highest at 37°C (significant). **D. *A. marginale* levels based on quantitative PCR for *msp5* in the midgut (green):** The infection level in midguts was highest at 37°C (significant) and lower at 4°C-22°C. P values show statistical relationship between temperature and quantity of *A. marginale* in panel B (analyzed as a continuous variable), while letters indicate statistical differences between individual treatments (analyzed as categorical variables) shown in panels C and D.

## Description

Ticks are blood feeding ectoparasites which biologically transmit viral, bacterial, and protozoal pathogens to humans and animals (Schwartz AM, 2017; Jacob et al., 2020; Wikel, 2018). Despite the significant health and economic impact of ticks, methods to control and prevent tick-borne diseases are limited. A primary reason for this is the large knowledge gaps in our understanding of basic tick physiology, particularly their thermal biology and the impact this has on vector competence. Ectothermic vectors cannot regulate their body temperatures so environmental temperatures strongly influence their survival, feeding, as well as the development and transmission of the pathogens they carry (Jacob et al., 2020). For example, temperature drives both pathogen development and transmission rates across geographic regions in mosquitoes (Shocket et al., 2020). Temperature can alter survival, growth, behavior, and geographic distributions of ticks, but it is poorly understood how pathogens influence these relationships (Burtis et al., 2016; Nielebeck et al., 2023; Qviller et al., 2014). *Ixodes scapulars* ticks infected with *Anaplasma phagocytophilum* expressed an antifreeze glycoprotein (IAFGP) that enhances tick survival in the cold, which may facilitate faster northward range shifts than climate change models predict (Neelakanta et al., 2010). Thus, it is important to understand the complex interactions between temperature stress in ticks and the pathogens they transmit, to predict the dynamics of pathogen transmission and determine risk across geographic regions.

*Anaplasma marginale* causes bovine anaplasmosis, one of the most common tick-borne diseases of cattle worldwide. Infection results in anemia, production losses, and, in some cases, unexpected high mortality outbreaks (Railey and Marsh, 2021; Kocan et al., 2003). *Anaplasma marginale* is endemic in much of the United States and is primarily transmitted by *Dermacentor* spp. ticks in the United States and Canada (Howden et al., 2010; Scoles et al., 2007). Though transstadial transmission is possible, intrastadial transmission is most common as male ticks move between cattle searching for mates and feed on multiple hosts (Kocan and de la Fuente, 2003; Kocan et al., 2010). *Anaplasma marginale* is an obligate, intracellular bacterium and thus must invade, and establish a protected niche in the tick cells to acquire nutrients and replicate (Magunda et al., 2016). During the first blood meal or acquisition feed, bovine erythrocytes infected with *A. marginale* enter the tick midgut epithelial cells and replicate in the tick. The bacteria transit to, invade and replicate in the salivary glands. After the blood meal, ticks fall off and look for another host. When the tick ingests a second blood meal, the bacteria are released into the salivary gland secretions, and transmission occurs (Darby et al., 2007; Rodríguez et al., 2009; Kocan et al., 2009). Importantly, during the transmission period, both the tick vector and *A. marginale* are off host and therefore exposed to environmental temperatures. The impact of temperature on the replication rates and colonization of *A. marginale* in ticks is unknown. Because the colonies of ticks used in this study were maintained at 26°C for several generations, we predicted that tick survival and *A. marginale* levels would be highest at this temperature and decline as temperature increased or decreased.

We measured the impact of temperature on replication rates of *A. marginale* in midguts and salivary glands of adult male *D. andersoni.* One calf was infected intravenously with the Saint Maries strain of *A. marginale* and monitored for infection using packed cell volume (PCV) as a measure of anemia and Giemsa-stained blood smears to count percent infected erythrocytes (PPE). ( Figure 1A ). Ticks were applied during rising infection as indicated by the PPE ( Figure 1A ). Adult *D. Andersoni* male ticks (n =117) fed on the infected calf for 7 days. All the ticks (n=117) were infected with *A. marginale* following feeding. The mean level of *A. marginale* at 32 hours post-feeding was 260K and 442Kcopies in midguts and salivary glands respectively (n=12). Immediately following feeding, the replete male ticks were placed into separate incubators at 4°C, 10°C, 15°C, 20°C, 22°C, 26°C, 32°C, 37°C, and 42°C for 7 days. Seven days as an incubation period was selected to give maximum time for bacterial replication while being short enough to prevent significant tick mortality. After feeding, adult male ticks begin to degrade and dissection of organs becomes more difficult as time since feeding increases (G. Scoles, pers comm., February 2025). After inoculation of *A. marginale* in calf, it takes 24-26 days for the development of Anaplasmosis. To ensure ticks fed during the highest period of bacteremia, we applied ticks approximately 7 days prior to Anaplasmosis, as defined by high levels of infected erythrocytes (Noh Susan et al., 2016; Löhr et al., 2002; Kocan et al., 1992; Kocan et al., 1993). After 7 days of temperature treatment, ticks were assessed for survival. All ticks exposed to 42°C died, and 2 ticks exposed to 37°C died. All ticks exposed to lower temperatures survived.

We then dissected tick midguts and salivary glands from all remaining ticks and measured the *A. marginale* levels in each tick tissue using quantitative PCR for *msp5* . *Msp5* is a single copy gene, and thus suitable to quantitate *A. marginale* levels in tick organs. Overall, the bacterial number significantly increased with temperature in both midguts and salivary glands (GLM: *F*_2,231_ =27.2, *P* <0.0005). Across all temperatures, infection tended to be higher in the salivary glands compared with midguts ( *F*_2,231_ =27.2, *P* <0.0005). *A. marginale* levels did not significantly vary between tick midguts exposed to treatments between 4°C-22°C and averaged 700K copies (Fig1D). However, bacterial levels were significantly higher at both 26°C and 32°C and averaged ~2.3M copies ( *F*_2,220_ =21.2, *P* <0.0005). The level of *A. marginale* in midgut was highest at 37°C (Fig1D).

In salivary glands, the *A. marginale* level was ~600K in ticks held between 4°C and 22°C and was ~19M copies in ticks held from 26°C to 37°C ( *F*_1,232_ =13.5, *P* <0.002). There was no significant difference in *A. marginale* levels in salivary glands of ticks held between 4-20°C (~190K; Fig 1C; *F*_7,109_ =9.3, *P* >0.05), but levels were significantly higher in ticks held at 26°C (~10M; *F*_7,109_ =9.3, *P* <0.043) and at 32°C (~13M; *F*_7,109_ =9.3, *P* <0.015). Similar to the midguts, infection levels in salivary glands were highest at 37°C increasing to approximately 35M copies ( *F*_7,109_ =9.3, *P* <0.00005). Overall, there was a 13-fold increase in *A. marginale* levels between tick midguts measured at 32hrs post feeding (~260k) and those measured after treatment at 37°C for 7 days (~3M). In the salivary glands, there was a 75-fold increase from 442k copies measured 32 hours post-feeding to 34M copies following treatment at 37°C for 7 days.

The general relationship between temperature and the amount of *A. marginale* in midguts and salivary glands was determined using smoothed curves (Fig 1B). We tested linear models with and without quadratic terms. Based on R ^2^ values, more variance was explained using a quadratic term for salivary glands (Linear model: F7,115=38.03, P<0.00005; R ^2^ =0.242; Quadratic model: F7,114=30.36, x:P=0.017, x ^2^ :P<0.0005; R ^2^ =0.3361), but a linear model was a better fit for the midguts (Linear model: F7,115=17.86, P<0.00005; R ^2^ =0.1269; Quadratic model: F7,114=10.87, x:P=0.4428, x ^2^ :P<0.0641; R ^2^ =0.1055). Our results suggest that *A. marginale* does not efficiently replicate in either midguts or salivary glands, off host, below 20°C and the ticks succumb at 42°C. From 22-37°C replication in the salivary glands and/or movement of *A. marginale* from the midgut to salivary glands increases significantly, possibly leading to increased probability for transmission during tick refeeding or an increased infectious dose delivered to the animal. Below 22°C, there is little to no replication of *A. marginale* in midguts or salivary glands. Whether cold-induced suppression of *A. marginale* replication in the salivary glands of ticks can lead to inhibition of transmission at lower temperatures remains unknown. Our findings could have important implications for the rate of replication of *A. marginale* in ticks across different environmental temperatures, which may influence transmission rates and doses of bacteria delivered to naïve animals. Because we only tested ticks at 7 days post feeding, more data is necessary to understand how differences in temperature may impact the viable transmission period for male *D. andersoni* , which can last up to 20 days (Noh et al., 2016).

Although little is known about the infectious dose for *A. marginale* , *D. andersoni* can successfully transmit to naïve cattle at levels of 10 ^4.1^ in the salivary glands (Scoles et al., 2008). The minimum dose of *A. marginale* in tick saliva required to infect cattle is unknown but should be tested in the future. Our study shows that *D. andersoni* can survive across broad temperature ranges (4-37°C), but experiences 100% mortality at 42°C. The high survival of both *A. marginale* and its vector across broad temperature ranges could explain the broad geographic distribution of this pathogen particularly in high latitude regions where winter temperatures inhibit survival of other tick species.

Tick and *A. marginale* survival throughout winter is especially critical for pathogens that cannot be passed from an infected adult female tick to its their offspring (Neelakanta et al., 2010). When pathogens are maintained in ticks throughout the year, their developmental stages are exposed to a variety of temperature changes which may slow or accelerate pathogen replication. Understanding how temperature impacts ticks and pathogens is a critical step towards assessing the global impact of climate change on ticks and tick-borne diseases. Here we show that *A. marginale* replication and persistence in the tick vector is temperature-mediated and may therefore be impacted by changes in environmental temperatures.

## Methods

**Experimental outline**

All animal use protocols were approved by the University of Idaho Institutional Animal Care and Use Committees (ASAF numbers 2732 and 2022-38). Field collected ticks were used to establish laboratory colonies, which were maintained at the Holm Research Center, USDA, ARS Animal Disease Research Unit in Moscow, Idaho and used for this study. One approximately 5-month-old Holstein calf was inoculated intravenously with 1.8ml of stabilate from the St. Maries strain of *A. marginale* and 4ml of its own serum (Eriks et al., 1994). Infection was tracked by light microscopic examination of Giemsa-stained blood smears to enumerate the percent of *A. marginale* -infected erythrocytes and packed cell volume ( Figure 1A ). One hundred adult male *D. andersoni* ticks were applied to the calf when percent infected erythrocytes reached 1.3% allowed to feed for 7 days (Kocan et al., 1992). Following removal, ticks were divided into groups of 11 and placed in incubators at 93% RH (Relative humidity), and 12:12: LD (Light:Dark) at either 4°C, 10°C, 15°C, 20°C 22°C, 26°C, 32°C, 37°C or 42°C for 7 days.

**DNA extraction**

Following the 7-day temperature exposure, midguts and salivary glands were dissected and stored in 225uL of ATL (a tissue lysis) buffer + 25uL of Proteinase K at –20°C instead of 200 uL (180 uL ATL buffer+ 20 uL Proteinase K) used in user protocol (Protocol, 2006). Genomic DNA was extracted using Qiagen DNeasy Blood and Tissue Kit (DNeasy kit, Qiagen, Hilden, Germany) following the manufacture’s guideline with the exception of 2 additional washes. DNA was eluted with 50 µl AE buffer.

**TaqMan quantitative PCR**

We enumerated genome copies of *A. marginale* per midgut and salivary gland using qPCR for *msp5* , a single copy *A. marginale* gene (Visser et al., 1992; Futse et al., 2003). Following primer sequences (forward 5’-CTTCCGAAGTTGTAAGTGAGGGCA-3′; reverse 5′-CTTATCGGCATGGTCGCCTAGTTT-3′) and a TaqMan probe (/56-FAM/CCTCCGCGT/ZEN/CTTTCAACAATTT GGTT/3IABkFQ/) were used to amplify the target gene (202 bp fragment).Each of 20 µl reaction was performed using Sso Advanced Universal Probes Supermix, 10 µM of each primer, 100 µM of TaqMan probe, and 2 µl of template DNA. Thermocycling conditions consisted of 95 °C for 3 min, followed by 40 cycles of at 95 °C for 15 sec, and annealing at 65 °C for 45 sec. The assay was performed using a Biorad CFX real-time PCR detection system (Bio-Rad Laboratories, Hercules, CA). The dilutions containing 10 ^8^ -10 ^1^ copies of plasmid DNA cloned with a *msp5* fragment were used for the standard curve. Quantitative PCR was conducted using triplicate operation for each sample. The average of three replicates was used in the data analysis.

**Statistical analysis**

The impact of temperature on *A. marginale* replication was tested with linear regression and emmeans package in R using untransformed data (Lenth, 2020). Levels of *A. marginale* was analyzed as a fixed effect with temperature (both as categorical and continuous variables) and tissue (midgut or salivary glands) as predictors in linear and quadratic models. We tested models with temperature as a continuous variable with and without quadratic terms. For global models to test overall effect of temperature on both midguts and salivary glands we pooled all samples, resulting in sample sizes up to 234 (117 ticks x 2 tissue types). Results for temperature treatments as categorical variables are shown in panels 1C and 1D, while the results for temperature as a continuous variable are shown in panel 1B. Smooth curves for visualizations in Figure 1B were made with geom_smooth()` using method = 'loess' and formula = 'y ~ x' with default settings for generating confidence intervals. All temperature treatments were compared using *least-squares means* using the Tukey method ( *emmeans)* with the pairwise method and the temperatures as categorical variables. Data visualizations were performed on log transformed data using ggplot2 package. All tests were conducted using the R Software v. 4.4.0 (Team, 2021).
